# Fatal Effects from the Bee’s Sting

**Published:** 1883-07-20

**Authors:** 


**Fatal Effects from the Bee's Sting.**—Elsewhere in this number of the journal we print an extract from the Lancet, going to show that the sting of the bee occasionally proves fatal. An additional instance, if we may credit a press dispatch, occurred last week in the town of Milan, in Tennessee. The report is, that a woman was stung on the nose, and died in a few minutes. If it should really prove that in a number of cases death may fairly be imputed to the sting of the bee, the matter will be well worth investigating. Ordinarily, as is well known, only a local irritation is produced. If we concede that the mosquito can implant the *Filaria sanguinis hominis* in a man's blood, it seems not unreasonable to conjecture that the bite or sting of various animals not usually venomous may be made so on occasions of their having fed upon some septic or noxious substance. It is highly desirable that well-observed cases of this sort should be put on record.—*N. Y. Med. Record.*

The Editor of this Journal is cognizant of a case of death resulting from the sting of a bee. In this case, however, there was evidently an idiosyncracy, as the same party had narrowly escaped death on a former occasion from the same cause.—Ed. So. Med. Record.]

